# India’s tobacco control law: implementation of prohibition by educational institutions

**DOI:** 10.5588/pha.25.0013

**Published:** 2025-09-03

**Authors:** R. Singh, A.L Frank

**Affiliations:** Department of Environmental and Occupational Health, Dornsife School of Public Health, Drexel University, Philadelphia, PA, USA.

**Keywords:** cigarettes, chewable tobacco, pan masala, COTPA Act, 2003, Central Information Commission

## Abstract

In India, tobacco products are a leading cause of preventable diseases, including cancer. Because of the health risks to children, we explored compliance and implementation of tobacco control law in relation to educational institutes (EIs). We discuss an issue of accountability by an EI in relation to compliance regarding the prohibition of tobacco vendors in close proximity to the EI. We also discuss various studies highlighting poor implementation of the law prohibiting tobacco sales near to EIs. It is our recommendation that the tobacco control law in India should be strengthened to reduce preventable diseases, including cancer.

India, with its large population, needs to promote the prevention of cancers (approximately half of which can be prevented) by controlling risk factors, such as smoking. For this reason, we examined the legislation regarding the prohibition of tobacco sales in close proximity to schools/colleges. In 2003, India passed the Cigarettes and Other Tobacco Products (Prohibition of Advertisement and Regulation of Trade and Commerce, Production, Supply and Distribution) Act, 2003 (COTPA Act 2003).^1^ Some parliamentarians initially opposed the Bill and raised issues such as the ‘health benefit of tobacco’ and providing employment to tobacco growers and small shopkeepers.^2^ Employment caused by tobacco cannot stand, as the same does not stand for other narcotic drugs.^3^ With harm to health being scientifically proven, and the projections for a rise in cancer cases in India being blamed in part on the use of tobacco products, it was justified to create and strengthen the law regarding tobacco control.^4,5^ This was noted by the parliamentary committee on cancer which placed a major focus on tobacco control.^6^ The COTPA Act was made in response to the 43^rd^ World Health Assembly, where member states were urged to consider tobacco control strategies, with a specific focus on protecting children from exposure to tobacco use and discouraging the use of tobacco.^7^ Tobacco initiation in India is reported as occurring from age 8–15 years, making this focus all the more crucial.^8^ The COTPA Act considered the early age of initiation by Section 6 of the COTPA Act which dealt with educational institutions (EIs), where young people spend a large part of their early life. The Section 6 of the COTPA Act 2003 is as follows:

*No person shall sell, offer for sale, or permit sale of, cigarette or any other tobacco product- (a) to any person who is under eighteen years of age, and (b) in an area within a radius of one hundred yards of any educational institution.*

This element was well intentioned, but its implementation was decentralised with on-ground enforcement done by outsourcing it to schools and colleges themselves. The College/School/Headmaster/Principal or even a teacher is an authorised officer to impose and collect fine against the violation of Section 4 of the COTPA Act 2003, which deals with prohibition of smoking in all public places including EIs.^1,9^ Apart from this collection of fines, the schools and colleges were given two rather simple tasks. First, to put up a board prohibiting sale of tobacco within 100 yards of the EI. Second to erect boards within its premises to prohibit people from smoking/using tobacco within the premises. But the law itself, along with the rules, are unclear who is responsibile for ensuring that there is no presence of tobacco vendors within the 100 yards. This was the issue before the Central Information Commission (No. CIC/ADIMV/C/2023/131421 Order dated 24.10.2024) where a college in Delhi had, in response to an information query under the Right to Information Act, 2005, transferred the questions of presence of vendors to the Delhi Police instead of providing an answer itself.^10^ In that matter, it was pleaded by the first author who was the complainant in the matter, that the guidelines by the Ministry of Health and Family Welfare clearly state that *‘*The educational institution management should ensure that no tobacco products are sold inside the premises and in an area within 100 yards from the premises’.^11^ In a landmark decision, the Central Information Commission stated that the ‘complaint raises significant issue of public interest particularly of a very vulnerable section of the society, i.e., schools and colleges’ and recommended that proactive measures should be taken by the college in this case.

Other studies, such as one in the administrative precincts of New Delhi, also noted that schools did not universally follow the signboard compliance of the COTPA Act.^12^ Another study examined compliance by the Central Government-run institutions of national importance.^13^ The National Institutes of Technology (NITs), Indian Institutes of Management (IIMs) and other institutions, refused to provide information regarding their compliance with the COTPA Act 2003. Some of the institutions went further and litigated against providing this information up to a second appeals before the Central Information Commission, and did not provide information on fines collected, the presence of vendors, or even whether boards had been installed.^13^ Another study found a lack of compliance by central universities.^14^ Also, compliance was not universal for higher educational institutions in Delhi.^15^ These reported cases highlight that something as simple as installation of notice boards prohibiting smoking, or prohibiting sales within 100 yards of an EI were not installed.

These findings raise doubts about the well-intentioned objective of Parliament to expect EIs to enforce tobacco control law, even though institutions are empowered to enforce compliance and the collection of fines. The COTPA Act, 2003 should be reinvigorated with strict accreditation barriers for EIs that do not comply with the law. Furthermore, the COTPA Act, 2003 currently has only mild punishments, e.g., the fine for smoking in public places and selling outside of EIs is only 200 rupees (USD 1–3). This is unlikely to serve as a deterrent or discourage tobacco use. Further steps, such as the amendment of the Juvenile Justice Act, 2015, which provides for stringent punishment for the sale of tobacco products to minors, are in the right direction. However, compliance with this law also needs to be evaluated and a focus must be on strengthening of the one single tobacco law to make it comprehensive and effective.^16^ Recent directions by the Delhi government for the appointment of officers to oversee compliance of the COTPA Act 2003 in schools, if not already done as per law, are welcome, but these will also need continuous follow up.^17^ This is vitally important as India, with its large population, needs to concentrate on promoting prevention of cancers by controlling risk factors. Among these, a leading risk factor is smoking.^18^
